# Autism Detection in Early Childhood (ADEC) in a Low-Income Spanish-Speaking Population in Guayaquil (Ecuador)

**DOI:** 10.1007/s10803-024-06413-3

**Published:** 2024-05-31

**Authors:** Susana Mata-Iturralde, Yurena Alonso-Esteban, Francisco Alcantud-Marín, Robyn Young

**Affiliations:** 1 Universidad de Guayaquil, Guayaquil, Ecuador; 2 Universidad de Zaragoza, Zaragoza, Spain; 3 Universidad de Valencia, Valencia, Spain; 4 Flinders University, Adelaide, Australia

**Keywords:** Autism Spectrum Disorder, Early Detection, ADEC, Adaptation, Reliability, Validation

## Abstract

Autism Spectrum Disorder (ASD) must be present early in development, but may not fully manifest until social demands exceed capacities. In the absence of adequate biological or brain imaging markers to detect and diagnose autism, diagnosis relies on clinical judgment based on observation of symptoms. Many tools have been developed in English-speaking countries (questionnaires for parents, symptom checklists for professionals, observation systems, etc.). Screening in countries with other languages requires cultural and linguistic adaptation of these instruments. This paper presents the adaptation of the ADEC (Autism Detection in Early Childhood).

**Methods**: The original version of the ADEC was translated and culturally and linguistically adapted to the characteristics of the population of Guayaquil (Ecuador).

**Participants**: A pilot study was conducted with a sample of 613 children aged 18–48 months. **Results**: Cronbach’s alpha values (0.89) indicate high internal consistency. The correlation between the MCHAT-R/F follow-up interview and the ADEC (mean *r* = 0.93) indicates high construct validity. In terms of predictive validity, using the original cut-off points of the ADEC, they show excellent diagnostic ability. The sensitivity and specificity results (sensitivity 1.00; specificity 0.92; positive predictive value 0.83; negative predictive value 0.99) are even better than those obtained in a similar study in the Mexican population. **Conclusions**: Considering that the MCHAT R/F is a parent-reported instrument, the Guayaquil Spanish version of the ADEC (ADEC-GU) seems to be a suitable instrument to be used in a complementary way as a second-level screening instrument for autism, before resorting to a full diagnostic process.

## Background

Autism Spectrum Disorder (ASD) is a neurodevelopmental condition characterized by a range of symptoms of varying severity that share significant impairments in communication and social interaction, and repetitive interests and behaviors. [Diagnostic and Statistical Manual of Mental Disorders, Fifth Edition, Text Revision (DSM-5-TR); (American Psychiatric Association [APA], 2022)].

In recent years, an upward trend in the prevalence of this disorder has been observed (Alcantud-Marin et al., 2016), partly explained by changes in diagnostic criteria, increased knowledge of the disorder, and more resources for detection and intervention. Currently, the prevalence of this disorder in the US is estimated at 1 in 36 children (2.8%) according to the most recent ADDM survey [Autism and Developmental Disabilities Monitoring, Centers for Disease Control and Prevention (CDC, 2023)]. In Australia, it is estimated to be between 0.6 and 1.3% depending on age (3–12 years) (Nielsen et al., 2023). In Europe, the upward trend is confirmed, albeit with a slightly lower prevalence (1–2% according to (Autism Europe, 2019). The global prevalence is estimated to be around 1% (Zeidan, y otros, 2022), although studies show great variability in estimates due to the use of different methodologies and socio-cultural and demographic variables. These data make ASD one of the most common neurodevelopmental disorders (Alcantud-Marin & Alonso-Esteban, 2022).

The causes of ASD are still largely unknown today, and no consistent biological markers or neurological images that can reliably detect risk or confirm a diagnosis have been identifies (Wang et al., 2015). The symptomatology of ASD affects the social, educational, and vocational development of individuals and their families, with varying degrees of severity throughout life.

However, there is evidence that early intervention (before the age of three) takes advantage of the plastic nature of early brain development, potentially minimizing the impact of the characteristic symptoms of ASD (Alcantud-Marin &Alonso-Esteban, 2O22, Dawson et al., 2012). This evidence, together with the increasing prevalence, justifies the need for early detection tools.

Although epidemiological studies show no differences by race or socioeconomic status (Baio et al., 2018), data from resource-poor countries are currently scarce, nonexistent, or methodologically questionable. In Ecuador, Dekkers et al. (2015) estimated the prevalence in Ecuador to be extremely low (0.11% diagnosed and 0.21% suspected). This study was conducted only in regular schools, and the authors themselves suspect bias due to intolerance of inclusion in regular schools. Fombonne et al. (2016) conducted a screening study in a Mexican population using the SRS [Social Responsiveness Scale (SRS); Constantino and Gruber (2005)] and found an average prevalence of 0.87%. There is some evidence that cultural and sociodemographic factors, particularly in low- and middle-income settings, may lead families to conceal symptoms of autism or to perceive them as “less severe” and not requiring professional support (Buffle et al., 2022). Underdiagnosis of ASD in low-resource countries (including Latin American countries) may also be due to the lack of translated and culturally adapted screening instruments and the cost of such instruments. (Alonso-Esteban et al., 2020; DuBay et al., 2023).The lack of early detection measures has the direct consequence that many children do not benefit from the outcomes of early intervention. Previous studies have analyzed the psychometric data of various screening instruments translated and adapted to the Spanish-speaking population. (Alonso-Esteban et al., 2020). The objective of this paper is to present the translation and cultural adaptation of the ADEC [Autism Detection in Early Childhood] (Young, 2007)] to the cultural reality of Guayaquil (Ecuador).

## Method

### Procedure

The adaptation of the ADEC was part of the project “Implementation of a Model for Early Detection of Autism Spectrum Disorder Risk in the MIES Programmes (CNH-CDI)” approved by the Ministry of Economic and Social Inclusion (Ref. MIES-SDIII-2021-0090-O of 11 August 2021) of Ecuador. This first phase consisted of selecting, translating and culturally adapting the screening instruments and piloting them in fourteen centers of the “Fundación Huerto de los Olivos”, a foundation that serves low-income families in a district of Guayaquil from January 2021 to September 2022 (Mata-Iturralde, 2023). This research is part of a larger project that was approved by the Ethics Committee in Human Research of the Universitat de València (Spain). The Code of Ethics of the World Medical Association (Declaration of Helsinki) was followed throughout the process.

**Fig. 1:**
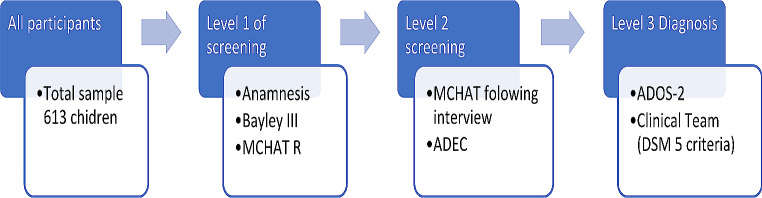
Graphical description of the steps followed in the screening data collection process

Following the recommendations and guidelines for the detection and diagnosis of ASD (Filipek et al., 2000; GAT, 2005), the assessment process began with an anamnesis interview in which information was requested about the gestation and birth of the child being evaluated and family history. Informed consent for the assessment of the child was also requested. At the same time, the Bayley III [Bayley Scales of Infant and Toddler Development; (Bayley, 2006)] was administered. During the time of administration of the developmental scale the parents completed the MCHAT-R/F (Modified Checklist for Autism in Toddlers – Revised with Follow (Robins et al., 2014). In a second interview, the follow-up interview of the MCHAT-R/F was administered. All children were assessed with the ADEC-GU and those at risk (preterm birth, siblings of autistic children, developmental delay, etc.) and those who tested positive were assessed with the ADOS-2. Finally, an independent clinical team made a diagnosis based on the Diagnostic and Statistical Manual of Mental Disorders.: Fifth Edition (American Psychiatric Association [APA], 2013) (see Fig. 1).

### Participants

Guayaquil is located on the Pacific coast of Ecuador and has a population of almost three million, of which 32.2% live in poverty and 14.7% in extreme poverty (INEC, 2021). Families attending the Child Development Centers of the “Huerto de los Olivos” Foundation in Guayaquil with children between 12 and 48 months of age were invited to participate. This foundation has 14 centers distributed throughout the city, mainly from the middle and lower social classes. A total of 613 children (49.75% girls, 50.25% boys) completed the evaluation process (see Table 1). The mean age of the fathers was 32.3 years (SD = 7.97) and of the mothers 29.33 years (SD = 7.12).

**Table 1:** Distribution of the sample for age and gender

*SEX*				*Mother’s age*		*Father’s age*	
**Age in months**	**Male**	**Female**	**Total**	**Mean**	**SD**	**Mean**	**SD**
Less than 18 months	44	60	104	29.55	7.53	32.48	32.48
19–24 months	20	23	43	26.56	6.10	29.07	29.07
25–30 months	33	33	66	30.50	7.26	34.03	34.03
31–36 months	63	55	118	28.59	6.54	31.79	31.79
37–42 months	99	79	178	29.71	7.33	32.51	32.51
43–48 months	49	55	104	29.70	7.08	32.30	32.30
Total	308	305	613	29.33	7.12	32.26	32.26

SD Standard Deviation

Mother and father’s age during gestation

### Measures

Following international recommendations (Filipek et al., 2000; GAT, 2005), a sequential screening process was designed consisting of three levels.

Level I: An ad hoc interview was designed to obtain information on the health history of the child and family. This anamnesis was complemented with the application of a developmental scale such as the Bayley Scales of Infant and Toddler Development (Bayley-III; Bayley, 2006), which assesses the development of infants and children aged 1 to 42 months. The Bayley-III has five subscales: Cognitive, Language, Motor, Social-Emotional, and Adaptive Behavior. The English version (Bayley, 2006) has high reliability coefficients (0.87-0.93). The Spanish adaptation of the Bayley-III scales (Bayley, 2015) was used in this study.

Level II: As a specific tool, the parent-reported MCHAT (Modified Checklist for Autism in Toddlers; Robins et al., 1999, 2001) is designed as a parent assessment questionnaire for children aged 16 to 30 months. Because of its ease of administration and accessibility, numerous studies have been conducted in different cultures, making it a worldwide standard. The version used in this study (MCHAT R / F; Robins et al., 2014), consists of 20 dichotomous response items (yes/no). It is administered in two phases, in the first phase parents answer the questionnaire and in the second phase, it includes a follow-up interview procedure for positive or doubtful results. There are different versions in Spanish (Canal et al., 2011; Manzone, 2013; Albores-Gallo et al., 2012; Coelho-Medeiros et al., 2017). Garcia Primo et al., (2014) have administered the M-CHAT to two general population samples at 18 and 24 months respectively. The results (S = 0.82-0.81; SP = 0.99-0.99; PPV = 0.38-0.43; NPV = 0.99-0.99) confirm the goodness of fit of the instrument when used in Spanish-speaking population. However, there is some controversy in the comparison of the results with the Hispanic population in the U.S. (Kimple et al., 2014) so in this study, we proceeded to conduct a review of the MCHAT R/F-SP version (Magán-Maganto et al., 2020) with the objective of adapting it to the population of Guayaquil.

The Autism Detection in Early Childhood (ADEC; Young, 2007; Young & Nah, 2016) is a level II observational screening instrument, applicable to children from 12 months of age. The age range of this test and the fact that it is uses direct observations make it a candidate to be a complementary test to others based on parent questionnaires whose results may be more biased by cultural and socio-demographic issues. It is composed of 16 items; each item involves observation of a reaction of the child being assessed during a play activity specifically designed to identify signs of autism. It will be scored as 0 (typical development), 1 or 2 (impact level of possible autistic behavior). The specific behaviors observed are: (1) name response, (2) imitation, (3) ritual play, (4) joint attention and social referencing, (5) eye contact, (6) functional play, (7) pretend play, (8) smile reciprocity, (9) reaction to common sounds, (10) gaze following, (11) following verbal commands, (12) delayed language, (13) anticipation of social advances, (14) nesting, (15) use of gestures, and (16) task switching. According to the sensitivity and specificity data provided in the manual (Young, 2007), a score of 0–10 indicates a low likelihood, a score of 11–13 a moderate likelihood, 14–19 a high likelihood and > 19 a very high likelihood of ASD. The ADEC (Young, 2007) in its English original version presents good psychometric data, in particular, high internal consistency (Cronbach’s α ranging from 0.85 to 0.93), test-retest reliability (*r* = 0.83) and inter-rater reliability (intraclass correlation coefficient, ICC = 0.83).

In other studies, it has also been shown to be robust. Thus, Nah et al. (2014a) found the ADEC to be a reliable and valid screening tool in comparison to other diagnostic instruments such as the ADOS (Lord et al., 1995), the ADI-R (Rutter et al., 2009) and the DSM-IV-TR (American Psychiatric Association [APA], 2000). In the same study (Nah, Young, Brewer, & Berlingeri, 2014a) they report high sensitivity (1.0), specificity (0.89) and predictive values (PPV = 0.84, NPV = 1.0) when using a cut-off score of 11. In subsequent studies, applying DSM-5 criteria (American Psychiatric Association [APA], 2013), Davis et al. (2015) obtain similar results (sensitivity 0.95; specificity 0.75; PPV 0.84; NPV 0.92). In follow-up studies (Nah et al., 2014b), the ADEC shows similar predictive values to other instruments such as the CARS (Schopler et al., 1986) both in predicting long-term outcomes and clinical diagnostic outcome and level of global adaptive functioning. There is a version translated into Spanish in Mexico. (Hedley, Young, Angelica, Gallegos, & Marcin, 2010) with very good psychometric results (Cronbach’s α = 0.73), adequate concurrent validity when contrasted with CARS (Schopler et al., 1986) and ADI-R (Rutter et al., 2009) and very appropriate validity Inter judges (0.96) (Hedley et al., 2010). These results were consistent with those obtained both in the original version and in other studies on the English population (Hedley et al., 2015; Nevill et al., 2019).

Level III: As a diagnostic support tool (Gold standard), we used the Autism Diagnostic Observation Schedule-2 (ADOS-2; Lord et al., 2012). The ADOS-2 is an evolution of the ADOS (Lord et al., 1995) and consists of a standardized, semi-structured observation of individuals who may belong to the autism spectrum. The activities are organized in five modules aimed at children and adults according to their developmental and language levels. In all of them different domains related to autistic behaviors are assessed (communication, reciprocal social interaction, communication & social interaction, imagination/creativity and stereotyped behaviors and restricted interests). Participants in this study were assessed with Module T or Module 1 or 2 according to their verbal fluency and age. For score calculation, we used the original algorithm proposed by Lord et al. (2012). Finally, all the information collected was used by clinicians to make a diagnostic judgment.

### Translation and Cultural Adaptation

The characteristics of the Spanish spoken by the population of Ecuador (Guayaquil), especially the population with low economic and cultural resources (Haboud & de la Vega, 2008), made it necessary to adapt the version of the instruments used. The translation and adaptation of the ADEC to the population of Guayaquil followed international standards. (Hambleton et al., 2005; Muñiz et al., 2013). A direct translation of the English version was done by a bilingual psychologist. Two focus groups were conducted to confirm terminology and wording more appropriate to the culture of Guayaquil:

The first focus group consisted of ten mothers of children under the age of four, who were asked to select the option they understood best for each item. The options presented corresponded to the Mexican ADEC-SP version and the direct translation version. All participants selected the item options that corresponded to their direct translation version, except for item 4, where they preferred to refer to the toy as “a toy that makes a noise that frightens or surprises the child” as in the Mexican version, rather than “a toy that bounces or is electronic”.

The second group consisted of seven professionals: two clinical psychologists, two educational psychologists, one occupational therapist, one speech therapist, and one English teacher. They were given the same instructions as the mothers’ focus group. For each item, they had to select the option corresponding to the Mexican version, their own version, and the version suggested by the mothers’ results.

Prototypes of the ADEC-GU were developed, incorporating the improvements agreed upon in the focus groups. It was then administered in the form of a survey in which 99 people between the ages of 20 and 50 participated (72.6% F; 27.4% M). For each of the 16 items, the participants had to choose the option that they understood best. The focus group modified version was more acceptable (65%) than the direct translation version (35%). With the help of the expert group, compliance with the quality control list for translation adaptation was checked (Hambleton et al., 2005; Hambleton & Zenisky, 2011; Muñiz et al., 2013). Finally, a back-translation into English was performed to ensure that there was no deviation in the meaning of the items and instructions. The format of the items is the same as the original version, with the same length of statements and response alternatives. The scripts and instructions reflect the same idea as the original version. The complete ADEC protocol in Spanish for Guayaquil is presented as supplementary material to this article.

## Results

According to the results of the developmental assessment (Bayley III), 90.54% of the children showed normative development, 3.26% had a medium probability of having a developmental disorder, and another 6.20% had a high probability of having a developmental disorder. In parallel, the results of the MCHAT R showed that 89.72% of the children assessed were not at risk of developing autism, compared to 8.32% with a moderate likelihood and 1.96% with a high likelihood. Given the objective of this study, the MCHAT follow-up interview and the ADEC were applied to all children, and the follow-up interview confirmed 98.69% of the cases, while the ADEC confirmed 92.82% of the cases.

To confirm the diagnosis, the ADOS-2 was administered to all children suspected of having autism, either because of biological risk (premature birth, siblings of other autistic children, etc.) or because they were positive on one of the instruments used. The group consisted of 86 children, of whom 63 were identified by the MCHAT and 55 were confirmed in the follow-up interview (see Table 2), but only 23 had a diagnosis confirmed by the ADOS-2 and the clinical team.

**Table 2:** Comparison of results between the MCHAT R/F and the ADEC

	MCHAT *R*/F		
ADEC	Negative	Positive	Total
Negative	558	36	594
Positive	0	19	19
**Total**	**558**	**55**	**613**

Chi-squared 198.83 degrees of freedom 1 *p* < 0.0001

Correlation 0.93 ** *p* < 0.001

In summary (see Table 3), the evaluation process was completed with a sample of 613 children, of whom 23 were diagnosed with autism (3.75%). It is noteworthy that 100% of the cases detected by ADEC (19 in total) were confirmed by ADOS-2 and by clinical diagnosis, while 4 children were not detected. Further research is needed, particularly to follow the development of children identified as autistic before the age of 24 months, in order to confirm these data.

**Table 3:** Distribution of diagnostic results according to the age of the participants

Diagnosis						
Age in months	No Diagnosis	ASD	GDD	ID	LD	Total
Less than 18 months	76	7	12	-	6	104
19–24 months	38	0	3	4	1	43
25–30 months	58	4	2	1	1	66
31–36 months	109	3	1	1	4	118
37–42 months	168	5	0	4	1	178
43–48 months	97	4	1	0	2	104
**Total**	**546**	**23**	**19**	**10**	**15**	**613**

GDD Global Developmental Disorder

ID Intelectual Disability

LD Language Disorders

### Internal Consistency

Conventionally, Alpha coefficient (Cronbach, 1951) is used as an indicator of internal consistency. As can be seen in Table 4, the ADEC-GU results show high internal consistency across all age cut-offs and the whole sample.

**Table 4:** Cronbach’s Alpha coefficient as a function of age levels

Age in months	Alpha
Less than 18 months	0.83
19–24 months	0.64
25–30 months	0.85
31–36 months	0.96
37–42 months	0.89
43–48 months	0.93
Total	0.89

The alpha indices indicate a good or very good internal consistency of the items that make up the ADEC-GU, both in the total sample and when segmented by age group. The maximum value (0.96) is reached in the group of children (31–36 months) and the lowest value in the group of 19–24 months (the group with the smallest number of children tested).

### Concurrent Validity

The correlation between the scores obtained when administering the MCHAT-R/F and the ADEC-GU was calculated for the entire sample, since the former is an instrument with proven validity. Table 5 presents the Pearson correlation coefficients between the scores, which show a significant value above 0.90. The ADEC-GU score is equivalent to the score obtained from the MCHAT-R questionnaire plus confirmation in the follow-up interview.

**Table 5:** Correlation coefficient between MCHAT-R/F total score and ADEC

Age in months	*r*_MCHAT/RF ADEC_
Less than 18 months	0.93**
19–24 months	0.89**
25–30 months	0.95**
31–36 months	0.92**
37–42 months	0.95**
43–48 months	0.93**
Total	0.93**

** Significance *P* > 0.01

### Predictive Validity

The ADEC manual (Young, 2007; Young et al., 2022) suggests that scores below 10 points can be considered as low likelihood, moderate from 11 to 13, high from 14 to 19 and very high above 19.

Considering that the selected, adapted and applied instruments are for the early detection of ASD, the ADOS-2 diagnostic test (Lord et al., 2012) was used as a gold standard. Different modules of the ADOS-2 were applied to 86 children identified with moderate or high likelihood by the other instruments and to children at risk at birth, confirming the diagnosis of ASD in 23 children. The correlation between the ADOS-2 score and the ADEC-GU was 0.91.

**Fig. 2:**
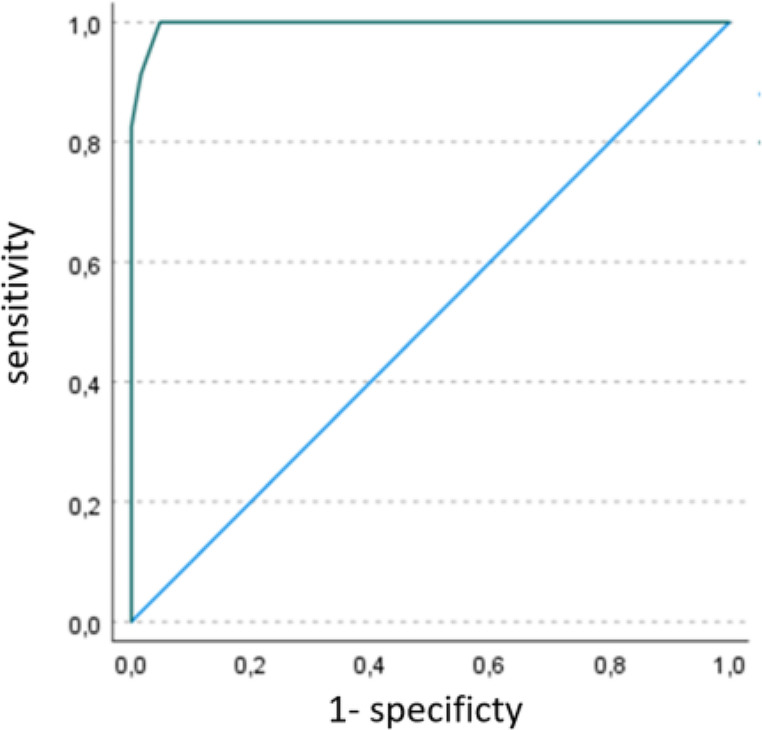
Curve ROC for ADEC detection

In order to explore the ability to discriminate, the Receiver Operating Characteristic (ROC) curve was used (see Fig. 2). The area under the curve (AUC) was also analyzed to determine the overall predictive validity index (AUC = 0.997). In addition, the optimal cut-off point was calculated, considering that, for screening tools, it is preferable to choose a cut-off point with a higher sensitivity. The more the curve is above the reference line, the more accurate the instrument will be and the closer the area under the curve is to 1.00, the higher the accuracy. (Zweig & Campbell, 1993). The predictive values obtained from the ADEC-GU (see Table 6 and 7) are similar to the study adapting the ADEC to Mexico, Hedley et al. (2010) obtained (Se = 0.79–0.94 and Spe = 0.88-1.0). In studies of the original English version Young et al. (Young et al., 2007; Nah et al., 2014a) report a sensitivity equal to 1.0 and a specificity between 0.74 and 0.90.

**Table 6:** Distribution of the number of children detected by the ADEC with low, moderate, high or very high risk of autism

Likelihood of ASD	Number	%
Low	594	96.90
Moderate	13	2.12
High	3	0.49
Very high	3	0.49
Total	613	100.0

**Table 7:** Comparison of MCHAT and ADEC results

	Sensitivity	Specificity	PPV	NPV	Precision
M-CHAT-R	1.00	0.93	0.37	1.00	0.93
M-CHAT-R/F	1.00	0.95	0.42	1.00	0.94
ADEC	0.88	1.00	0.95	0.99	0.99

### Discussion and Conclusion

The prevalence of autism spectrum disorders in Ecuador is unknown and even less so in the city of Guayaquil. Therefore, we cannot know if the results obtained reflect true prevalence or are an artifact of the detection process. Based on the data obtained, the prevalence of ASD in Guayaquil could be estimated at around 3.75% of children between 18 and 48 months of age. The age of administration influenced the number of cases detected, and a possible bias in the composition of the sample in our study (parents participated voluntarily and it is therefore possible that more parents attended if they had any suspicions). There is evidence to suggest the possibility of a higher false positive rate below two years of age and especially before 18 months (Chawarska et al., 2007; Pandey et al., 2008). On the other hand, research suggests that children falsely identified as having autism before 18 months later presented with another neurodevelopmental disorder. Further research and case follow-up is needed to determine the percentage of false positives (Pandey et al., 2008; Pierce et al., 2011).

The use of a tool such as the MCHAT R/F in low-income countries is justified by its accessibility and low cost. However, low socioeconomic levels are associated with low levels of parental education and may lead to biases in their responses (Alonso-Esteban et al., 2020). Socio-cultural differences may underlie the differences in ASD prevalence rates (Tek & Landa, 2012). Evidence suggests that culture, education, and beliefs about the causes, course, and treatment of autism influence perceptions of the importance and severity of symptoms at the time of assessment (Mandell & Novak, 2005). The risk of lowering the screening age below 24 months, as discussed, is an increase in false positives. However, the benefit is the possibility of initiating earlier and potentially more beneficial intervention. The use of an observational tool such as ADEC as level II may improve the results of the screening process.

The aim of this paper is to present the version of the ADEC-GU for the population of Guyaquil (Ecuador) in response to the growing need for linguistically and culturally adapted instruments for the context of application. The interest in having different screening instruments for ASD is based on two principles: (a) the increase in prevalence and (b) the positive results of early intervention.

Although great efforts are being made internationally to develop new screening algorithms based on AI (artificial intelligence) (Moridian et al., 2022; Shahamiri & Thabtah, 2020), with the use of applications accessible through mobile devices such as smartphones, in low-resource countries, conventional instruments are still needed to be adapted accordingly. The ADEC is a level II screening tool, i.e., specifically designed for the detection of ASD, which operates by observation of the child’s behavior in different play and social interaction situations. It complements the use of other instruments based on questionnaires answered by parents, such as the MCHAT-R/F, which is more accessible and economical in its application but introduces cultural biases that are difficult to treat. The psychometric data of the ADEC show that it can become a low-cost complementary instrument that can be used by health personnel with a low level of training in ASD.

Given the need to take advantage of early detection to initiate intervention, different screening systems combining more than one instrument have been proposed (For example, Nygren et al., 2012; Harris & Norton, 2016).

The possibility of developing low-cost screening programs with accessible tools (e.g. Nygren et al., 2012; Harris & Norton, 2016) in which two instruments such as the MCHAT R/F and a second observational instrument such as the ADEC are combined facilitates screening while minimizing the risks of error. Further research and collection of follow-up data is needed to confirm both the prevalence of the disorder in Guayaquil and the goodness of fit of the instruments used.

### Study Limitations

Although the sample size is adequate, it would be useful to replicate the study in a general non-volunteer sample and from different socioeconomic strata. Also, as mentioned in the text, a follow-up is necessary to evaluate the cases of false positives and false negatives.

### Future Lines of Research

The cost of screening using a three-level system remains a limitation in countries such as Ecuador, with low resources. A cost/effectiveness study is needed to determine their usefulness. In this line, research should be done on the advantages of using abbreviated forms by professionals with low specialized training in order to reach the entire population. Finally, we believe that early detection only makes sense if there is the possibility of developing early intervention, so we should investigate the effects of early intervention programs implemented in Guayaquil (Ecuador).

## References

[CR1] Albores-Gallo, L., Roldan-Ceballos, O., Villarreal-Valdes, G., Betanzos-Cruz, B., Santos-Sánchez, C., Martínez-Jaime, M., & Hilton, C. (2012). M-Chat Mexican version validity and reliability and some cultural considerations. *International Scholarly Research Notices Article ID*, 408697. 10.5402/2012/408694. 10.5402/2012/408694 PMC3395208 22811934

[CR3] Alcantud-Marin, F., & Alonso-Esteban, Y. (2022). *Trastornos Del Espectro Del Autismo: Bases para la intervención psicoeducativa*. Piramide.

[CR2] Alcantud-Marin, F., Alonso-Esteban, Y., & Mata-Iturralde, S. (2016). Prevalencia De Los Trastornos Del Espectro Autista: Revisión De Datos. *Siglo Cero: Revista Española Sobre Discapacidad Intelectual*, *47*(4), 7–26. 10.14201/scero2016474426.

[CR4] Alonso-Esteban, Y., Marco, R., Hedley, D., Uljarevié, M., Barbaro, J., Canal-Bedia, R., & Alcantud-Marín, F. (2020). Screening instruments for early detection of autism spectrum disorder in Spanish speaking communities. *Psicothema*, *32*(2), 245–252. 10.7334/psicothema2019.340. 32249751 10.7334/psicothema2019.340

[CR5] American Psychiatric Association [APA]. (2000). *Diagnóstic and Statistical Manual of Mental Disorders.Text Revision (DSM-IV-TR)*. American Psychiatric Association.

[CR6] American Psychiatric Association [APA]. (2013). *Diagnostic and statistical Manual of Mental disorders* (5th ed.). American Psychiatric Publishing.

[CR7] American Psychiatric Association [APA]. (2022). *Diagnóstic and Statistical Manual of Mental Disorders. 5th T R Edition*. American Psychiatric Association.

[CR8] Autism Europe (2019). *Personas con Trastorno del Espectro del Autismo* Bruxeles: Autism Europe. Retrieved from https://www.autismeurope.org/wp-content/uploads/2019/11/People-with-Autism-Spectrum-Disorder.-Identification-Understanding-Intervention_Spanish-version.pdf.

[CR9] Baio, J., Wiggins, L., Christensen, D. L., Maenner, M. J., Daniels, J., Zachary, W., & Dowling, N. F. (2018). Prevalence of Autism Spectrum Disorder among children aged 8 years. *Autism and Developmental Disabilities Monitoring Network*, *67*(6), 1–23. 10.15585/mmwr.ss6706a1. 10.15585/mmwr.ss6706a1 PMC5919599 29701730

[CR10] Bayley, N. (2006). *Bayley-III: Bayley scales of Infant and Toddler Development*. Pearson.

[CR11] Bayley, N. (2015). *Spanish adaptation of the Bayley scales of Infant and Toddler Development* (Third ed.). Pearson Educación S.A.

[CR12] Buffle, P., Gentaz, E., & Vivanti, G. (2022). Perception, beliefs, and causal attribution of Autism Early signs in Ecuadorian General Population. *Frontiers in Psychology*, *13*, 915817. 10.3389/fpsyg.2022.915817. 35814115 10.3389/fpsyg.2022.915817 PMC9260421

[CR13] Canal-Badia, R., García-Primo, P., Martin-Cilleros, M., Santos-Borbujo, J., Guisuraga-Fernández, Z., Herráez-García, L., & Posada-de la Paz, M. (2011). Modified Checklist for Autism in Toddlers: Cross-cultural adaptation and validation in Spain. *Journal of Autism and Developmental Disorders*, *41*(10), 1342–1351. 10.1007/s10803-010-1163-z. 21161677 10.1007/s10803-010-1163-z

[CR14] CDC (2023). Autism Prevalence Higher, According to Data from 11 ADDM Communities: econd report highlights disruptions in early autism detection at the start of the COVID-19 pandemic. *Morbidity and Mortality Weekly Report (MMWR)*, 404, 639–3286. Retrieved from https://www.cdc.gov/media/releases/2023/p0323-autism.html.

[CR15] Chawarska, K., Klin, A., Paul, K., & Volkmar, F. (2007). Autism spectrum disorder in the second year: Stability and change in syndrome expression. *Journal of Child Psychology and Psychiatry*, *48*, 128–138. 10.1111/j.1469-7610.2006.01685.x. 17300551 10.1111/j.1469-7610.2006.01685.x

[CR16] Coelho-Medeiros, M. E., Bronstein, J., Aedo, K., Pereira, J. A., Arraño, V., Pérez, C. A., Valenzuela, P. A., Moore, R., Garrido, I., & Bedregal, P. (2017). Importance of cross-cultural adjustment of M-CHAT-R/F in the process of validation as an Autism Test. *Revista Chilena De Pediatría*, *88*(6), 822–823. 10.4067/S0370-41062017000600822. 29546936 10.4067/s0370-41062017000600822

[CR17] Constantino, J. N., & Gruber, C. P. (2005). *Social Responsiveness Scale (SRS)*. Western Psychological Services.

[CR18] Cronbach, L. (1951). Coefficient alpha and the internal structure of tests. *Psychometrika*, *16*, 297–334. 10.1007/BF02310555.

[CR19] Dawson, G., Jones, E., Merkle, K., Venema, K., Lowy, R., Faja, S., & Webb, S. (2012). Early behavioral intervention is Associated with normalized brain activity in Young Children with Autism. *Journal of the American Academy of Child and Asolescent Psychiatry*, *51*(11), 1150–1159. 10.1016/j.jaac.2012.08.018. 10.1016/j.jaac.2012.08.018 PMC3607427 23101741

[CR20] Dekkers, L., Groot, N., Díaz Mosquera, E., Andrade-Zúñica, I. P., & Delfos, M. F. (2015). Prevalence of Autism Spectrum disorders in Ecuador: A pilot study in Quito. *Journal of Autism and Developmental Disorders*, *45*, 4165–4173. 10.1007/s10803-015-2559-6. 26319251 10.1007/s10803-015-2559-6 PMC4653240

[CR21] DuBay, M., Lee, H., & Palomo, R. (2023). Evidence map of Spanish language parent- and self-report screening and diagnostic tools for autism spectrum disorder. *Research in Autism Spectrum Disorders*, 102117. 10.1016/j.rasd.2023.102117. 102.

[CR22] Filipek, P., Accardo, P., Ashwal, S., Baranek, G., Cook, E., Dawson, G., Gordon, B., Gravel, J. S., Johnson, C. P., Kallen, R. J., Levy, S. E., Minshew, N. J., Ozonoff, S., Prizant, B. M., Rapin, I., Rogers, S. J., Stone, W. L., Teplin, S. W., Tuchman, R. F., & Volkmar, F. (2000). Practice parameter: Screening and diagnosis of Autism: Report of the Quality standards Subcommittee of the American Academy of Neurology and the Child Neurology Society. *Neurology*, *55*(0028-3878), 468–479. 10.1212/wnl.55.4.468. 10953176 10.1212/wnl.55.4.468

[CR23] Fombonne, E., Marcin, C., Manero, A., Bruno, R., Diaz, C., Villalobos, M., & Nealy, B. (2016). Prevalence of Autism Spectrum disorders in Guanajuato, Mexico: The Leon survey. *Journal of Auitsm and Developmental Disorders*, *46*(5), 1669–1685. 10.1007/s10803-016-2696-6. 10.1007/s10803-016-2696-6 26797939

[CR24] Garcia-Primo, P., Santos, J., Martin, M., Martinez, M., Lleras, S., Posada de la Paz, M., & Canal, R. (2014). Programa De detección precoz de trastornos generalizados del desarrollo en las áreas de salud de Salamanca y Zamora. *Anales De Pediatría*, *80*(5), 285–292. 10.1016/j.anpedi.2013.06.030. 24103249 10.1016/j.anpedi.2013.06.030

[CR25] GAT. (2005). Guía De Buena práctica para El diagnóstico De Los trastornos del espectro autista [Best practice guidelines for the diagnosis of autistic spectrum disorders]. *Revista De Neurologia*, *41*(5), 299–310. 10.33588/rn.4105.2005057. 16138288

[CR26] Haboud, M., & de la Vega, E. (2008). El español en Ecuador. In, Palacios, A. (Ed.). *El español en América: Contactos lingüísticos en Hispanoamérica* (pp.161–187). Ariel Letras.

[CR27] Hambleton, R. K., & Zenisky, A. L. (2011). Translating and adapting tests for cross-cultural assessments. In D. En, Matsumoto, & van de F. J. Vijver (Eds.), *Cross-cultural research methods in psychology* (pp. 46–74). Cambridge University Press.

[CR28] Hambleton, R., Merenda, P., & Spielberger, C. (2005). *Adapting educational and psychological tests for cross cultural assessment*. Lawrence Erlbaum.

[CR29] Harris, J., & Norton, A. (2016). The Quick Peek Program: A model for Developmental Screening within Underserved communities. *Infants & Young Children*, *29*(4), 290–298. 10.1097/IYC.0000000000000073.

[CR31] Hedley, D., Young, R., Angelica, M., Gallegos, J., & Marcin Salazar, C. (2010). Cross-cultural evaluation of the Autism detection in early childhood (ADEC) in Mexico. *Autism*, *14*(2), 93–112. 10.1177/1362361309347676. 20395280 10.1177/1362361309347676

[CR30] Hedley, D., Nevill, R., Monriy-Moreno, Y., Fields, N., Wilkins, J., Butter, E., & Mulick, J. (2015). Efficacy of the ADEC in identifying Autism Spectrum Disorder in clinically referred toddlers. *Journal of Autism and Developmenatl Disorders*, *45*(8), 2337–2348. 10.1007/s10803-015-2398-5. 10.1007/s10803-015-2398-5 25737020

[CR32] INEC (Instituto Nacional de Estadística y Censo del Ecuador) (2021). Encuesta Nacional de Empleo, Desempleo y Subempleo 2021 (ENEMDU) Indicadores de Pobreza y Desigualdad. Retreibed from https://www.ecuadorencifras.gob.ec/documentos/web-inec/POBREZA/2021/Junio-2021/202106_PobrezayDesigualdad.pdf.

[CR33] Kimple, K., Bartelt, E., Wysocki, K., & Steiner, M. (2014). Performance of the modified checklist for Autism in toddlers in Spanish-speaking patients. *Clinical Pediatrics*, *53*(7), 632–638. 10.1177/0009922814522346. 24550559 10.1177/0009922814522346

[CR34] Lord, C., Rutter, M., & Le Couteur, A. (1995). *Autism Diagnostic Observation schedule (ADOS) manual*. Western Psychological Services.

[CR35] Lord, C., Rutter, M., DiLavore, P., Risi, S., Gotham, K., & Bishop, S. (2012). *Autism diagnostic Observation schedule, second edition: ADOS-2*. Western Psychological services.

[CR36] Magán-Maganto, M., Canal-Bedia, R., Hernández-Fabián, A., Bejarano-Martín, A., Fernández-Alvarez, C., Martínez-Velarte, M., Martín-Cilleros, M. V., Flores-Robaina, N., Roeyers, H., & de la Posada, M. (2020). Spanish Cultural Validation of the Modifi ed Checklist for Autism in toddlers, revised. *Journal of Autism and Developmental Disorders*, *50*(7), 2412–2423. 10.1007/s10803-018-3777-5. 30328577 10.1007/s10803-018-3777-5

[CR37] Mandell, D., & Novak, M. (2005). The role of culture in families’ treatment decisions for children with autism spectrum disorders. *Mental Retardation and Developmental Disabilities Research Reviews*, *11*(2), 110–115. 10.1002/mrdd.20061. 15977313 10.1002/mrdd.20061

[CR38] Manzone, L. A. (2013). Adaptación y validación del Modified Checklist for Autism in Toddler para población urbana argentina. *Psicodebate*, 12, 79–105. Retrieved from https://dialnet.unirioja.es/servlet/articulo?codigo=5645321.

[CR39] Mata-Iturralde, S. (2023). *Validación y Estandarización de un Sistema de Detección Temprana de Trastorno Espectro Autista como propuesta para ser implementado en el Sistema Nacional de Salud* Valencia: Universitat de Valencia. Retrieved from https://roderic.uv.es/handle/10550/88421.

[CR40] Moridian, P., Ghassemi, N., Jafari, M., Salloum-Asfar, S., Sadeghi, D., Khodatars, M., & Acharya, U. (2022). Automatic autism spectrum disorder detection using artificial intelligence methods with MRI neuroimaging: A review. *Frontiers in Molecular Neuroscience*, *15*, 99605. 10.3389/fnmol.2022.999605. 10.3389/fnmol.2022.999605 PMC9577321 36267703

[CR41] Muñiz, J., Elosua, E., & Hambleton, R. (2013). Guidelines for the translation and adaptation of the tests: Second edition. *Psicothema*, *25*(2), 151–157. 10.7334/psicothema2013.24. 23628527 10.7334/psicothema2013.24

[CR42] Nah, Y. H., Young, R. L., Brewer, N., & Berlingeri, G. (2014a). Autism detection in early childhood (ADEC): Reliability and validity data for a level 2 screening tool for autistic disorder. *Psychological Assessment*, *26*(1), 215–226. 10.1037/a0034472. 24490680 10.1037/a0034472

[CR43] Nah, Y., Young, R., & Brewer, N. (2014b). Using the Autism detection in early childhood (ADEC) and Childhood Autism Rating scales (CARS) to Predict Long term outcomes in children with Autism Spectrum disorders. *Journal of Autism and Developmental Disorders*, *44*, 2301–2310. 10.1007/s10803-014-2102-1. 24658894 10.1007/s10803-014-2102-1

[CR44] Nevill, R., Hedley, D., & Uljarević, M. (2019). Brief report: Replication and validation of the Brief Autism Detection in Early Childhood (BADEC) in a clinical sample. *Journal of Autism and Developmental Disorders*, *49*(11), 4674–4680. 10.1007/s10803-019-04153-3. 31372801 10.1007/s10803-019-04153-3

[CR45] Nielsen, T., Nassar, N., Boulton, K., Guastella, A. J., & Lain, S. (2023). Estimating the prevalence of Autism Spectrum Disorder in New South Wales, Australia: A Data linkage study of three routinely collected datasets. *Journal of Autism and Developmental Disorders*. 10.1007/s10803-022-05887-3. 36652127 10.1007/s10803-022-05887-3 PMC10981615

[CR46] Nygren, G., Sandberg, E., Gillstedt, F., Ekeroth, G., Arvidsson, T., & Gillberg, C. (2012). A new screening programme for autism in a general population of Swedish toddlers. *Research in Developmental Disabilities*, *33*(4), 1200–1210. 10.1016/j.ridd.2012.02.018. 22502846 10.1016/j.ridd.2012.02.018

[CR47] Pandey, J., Verbalis, A., Robins, D. L., Boorstein, H., Klin, A., Babitz, T., & Fein, D. (2008). Screening for autism in older and younger toddlers with the modified checklist for Autism in toddlers. *Autism*, *12*(5), 513–535. 10.1177/1362361308094503. 18805945 10.1177/1362361308094503

[CR48] Pierce, K., Carter, C., Weinfeld, M., Desmond, J., Hazin, R., Bjork, R., & Gallagher, N. (2011). Detecting, studying, and treating autism early: The one-year well-baby check-up approach. *The Journal of Pediatrics*, *159*(3), 458–465. 10.1016/j.jpeds.2011.02.036. 21524759 10.1016/j.jpeds.2011.02.036 PMC3157595

[CR49] Robins, D. L., Fein, D., & Barton, M. L. (1999). *Follow-up interview for the modified checklist for autism in toddlers (M-CHAT FUI)*. Self-Published.

[CR50] Robins, D., Fein, D., Barton, M., & Green, J. (2001). The modified checklist for Autism in toddlers: An Inicial Study Investigating the Early Detection of Autism and Pervasive Developmental disorders. *Journal Od Autism and Developmental Disorders*, *31*(2), 131–144. 10.1023/A:1010738829569. 10.1023/a:1010738829569 11450812

[CR51] Robins, D., Casagrande, K., Barton, M., Chi-Ming, A., Dumont-Mathieu, T., & Fein, D. (2014). Validation of he modified Checklist for Autism in toddlers, revised with folow-up (M-CHAT-R/F). *Pediatrics*, *133*(1). 10.1542/peds.2013-1813. 10.1542/peds.2013-1813 PMC3876182 24366990

[CR52] Rutter, M., Le Couteur, A., & Lord, C. (2009). *Autism diagnostic interview revised*. Autism Genetic Resource Exchange.

[CR53] Schopler, E., Reichler, R., & Renner, B. (1986). *The Childhood Autism Rating Scale (CARS): For diagnostic screening and classification of autism*. Irvington.

[CR54] Shahamiri, S., & Thabtah, F. (2020). Autism AI: A New Autism Screening System based on Artificial Intelligence. *Cognitive Computation*, *12*, 766–777. 10.1007/s12559-020-09743-3.

[CR55] Tek, S., & Landa, R. (2012). Differences in autism symptoms between minority and non-minority toddlers. *Journal of Autism and Developmental Disorders*, *42*(9), 1967–1973. 10.1007/s10803-012-1445-8. 22271196 10.1007/s10803-012-1445-8 PMC3402594

[CR56] Wang, Y., Wang, P., Xu, X., Godstein, J., McConkie, A., Cheung, S., & Jiang, Y. (2015). Genetics of Autism Spectrum disorders: The opportunity and challenge in the Genetics Clinic. In H. Fantemi (Ed.), *The molecular basis of Autism* (pp. 33–66). Springer. 10.1007/978-1-4939-2190-4.

[CR57] Young, R. (2007). *Autism detection in early childhood: ADEC*. Australian Council of Educational Research.

[CR59] Young, R., & Nah, Y. H. (2016). Examining autism detection in early childhood (ADEC) in the early identification of Young Children with Autism Spectrum disorders (ASD). *Austrlian Psychologist*, *51*(4), 261–271. 10.1111/ap.12223.

[CR58] Young, R., Brewer, N., Nah, Y. H., & Lim, A. (2022). *Autism Detection in Early Childhood: ADEC (2nd Edition)*. Australian Council of Educational Research (ACER Press).

[CR60] Zeidan, J., Fombonne, E., Scorah, J., Ibrahim, A., Durkin, M., Saxena, S., & Elsabbagh, M. (2022). Global prevalence of autism: A systematic review update. *Autism Research*, *15*, 778–790. 10.1002/aur.2696. 35238171 10.1002/aur.2696 PMC9310578

[CR61] Zweig, M. H., & Campbell, G. (1993). Receiver-operating characteristic (ROC) plots: A fundamental evaluation tool in clinical medicine. *Clinical Chemistry*, *39*(4), 561–577. 10.1093/clinchem/39.4.561. 8472349

